# Frequency, Progression, and Current Management: Report of 16 New Cases of Nonfunctional Pancreatic Neuroendocrine Tumors in Tuberous Sclerosis Complex and Comparison With Previous Reports

**DOI:** 10.3389/fneur.2021.627672

**Published:** 2021-04-09

**Authors:** Kate Mowrey, Hope Northrup, Peyton Rougeau, S. Shahrukh Hashmi, Darcy A. Krueger, Daniel Ebrahimi-Fakhari, Alexander J. Towbin, Andrew T. Trout, Jamie K. Capal, David Neal Franz, David Rodriguez-Buritica

**Affiliations:** ^1^Division of Medical Genetics, Department of Pediatrics, McGovern Medical School, University of Texas Health Science Center at Houston, Houston, TX, United States; ^2^Department of Pediatrics, Pediatric Research Center, McGovern Medical School, University of Texas Health Science Center at Houston, Houston, TX, United States; ^3^Division of Neurology, Department of Pediatrics, Cincinnati Children's Hospital Medical Center, Cincinnati, OH, United States; ^4^Division of Neurology, Department of Pediatrics, University of Cincinnati College of Medicine, Cincinnati, OH, United States; ^5^Department of General Pediatrics, University Children's Hospital Muenster, Muenster, Germany; ^6^Department of Radiology, Cincinnati Children's Hospital Medical Center, Cincinnati, OH, United States; ^7^Department of Radiology, University of Cincinnati College of Medicine, Cincinnati, OH, United States

**Keywords:** pancreatic neuroendocrine tumor, nonfunctional, tuberous sclerosis, surveillance, abdominal imaging

## Abstract

**Background:** Tuberous sclerosis complex (TSC) is a genetic condition that causes benign tumors to grow in multiple organ systems. Nonfunctional pancreatic neuroendocrine tumors (PNETs) are a rare clinical feature of TSC with no specific guidelines outlined for clinical management at this time. Our purpose is to calculate the frequency of nonfunctional PNETs as well as characterize the presentation, current clinical management, and assess the impact of systemic mammalian target of rapamycin (mTOR) on nonfunctional PNETs in TSC.

**Methods:** This retrospective chart review was performed by a query of the TS Alliance's Natural History Database and the Cincinnati Children's Hospital TSC Database for patients with nonfunctional PNET. Clinical data from these two groups was summarized for patients identified to have a nonfunctional PNET and compared to previously reported cases with TSC and nonfunctional PNETs.

**Results:** Our calculated frequency of nonfunctional PNETs is 0.65%. We identified 16 individuals, nine males and seven females, with a median age of 18.0 years (interquartile range: −15.5 to 25.5). Just over half (56.3%, *n* = 9) of the patients provided results from genetic testing. Six had pathogenic variants in *TSC2* whereas three had pathogenic variants in *TSC1*. The average age at PNET diagnosis was 15.0 years (range: 3–46 years). Almost all individuals were diagnosed with a PNET during routine TSC surveillance, 56.3% (*n* = 9) by MRI, 12.5% (*n* = 2) by CT, 25% (*n* = 4) by ultrasound, and 6.2% (*n* = 1) through a surgical procedure. Follow up after diagnosis involved 68.8% (*n* = 11) having serial imaging and nine of the sixteen individuals proceeding with surgical removal of the PNET. Eight individuals had a history of using systemic mTOR inhibitors. Tumor growth rate was slightly less in individuals taking an mTOR inhibitor (−0.8 mm/yr, IQR: −2.3 to 2.2) than those without (1.6 mm/yr; IQR: −0.99 to 5.01, *p* > 0.05).

**Conclusions:** Nonfunctional PNETs occurred at younger ages in our TSC cohort and more commonly compared to ages and prevalence reported for the general population. PNETs in patients on systemic mTOR inhibitors had lower rates of growth. The outcome of this study provides preliminary evidence supporting the use of mTOR inhibitor therapy in conjunction with serial imaging as medical management for nonfunctional PNETs as an alternative option to invasive surgical removal.

## Introduction

Tuberous sclerosis complex (TSC) is a rare, multi-system genetic condition that causes benign tumors to grow in the brain and in other vital organs, such as skin, kidneys, heart, lungs, and eyes. The estimated incidence of TSC is one in ~11,000 live births (1). The clinical presentation of TSC has significant inter- and intra-familial variability that leads to a spectrum of severity seen amongst affected individuals (2, 3). TSC is caused by mono-allelic pathogenic variants in *TSC1* or *TSC2*, which encode for hamartin and tuberin, respectively (4, 5) *TSC1* pathogenic variants causes 26% of the cases and *TSC2* pathogenic variants causes 69% of the cases. In general, *TSC2* pathogenic variants tend to be associated with a more severe clinical presentation and account for the majority of *de novo* cases of TSC (6). Surveillance recommendations for TSC were established in 2012 and called for all individuals with TSC to have routine brain MRIs to monitor for the emergence of subependymal giant cell astrocytomas (SEGA) and abdominal MRIs to monitor for the emergence and/or progression of kidney findings, which include renal cysts and angiomyolipomata (2). Serial abdominal imaging of this patient population has led to an increase in the identification of pancreatic masses that are presumed to reflect neuroendocrine tumors (PNETs) (7–12).

According to the recent 2019 WHO classification system, pancreatic neuroendocrine neoplasias (PNEN) are classified as either PNET or poorly differentiated Pancreatic Neuroendocrine carcinoma (PNEC) (13). These are two different entities are determined based on the degree of cellular differentiation (i.e., Ki-67 value) that present with different genetic etiologies, as well as clinical presentation, radiological features, treatment and prognosis (12, 14). Sporadic PNETs are extremely rare with an annual incidence of 0.43 in 100,000 (15–17). Ten percent of PNETs present in association with genetic syndromes including Multiple Endocrine Neoplasia Type I (MEN-1), Neurofibromatosis Type 1 (NF-1), TSC, and von Hippel-Lindau (VHL) (18–22). Tumor grading and staging are the two most important aspects that define management for PNETs. Depending upon their hormone production, PNEN can also be classified as functional or nonfunctional, with the former presenting earlier during the clinical course due to the symptoms associated with abnormal hormonal production and often require special molecular imaging techniques for localization (23). Insulinomas are the most common functional PNETs, followed by gastrinomas and VIPomas (12, 24). Nonfunctional tumors present later during their clinical course, and rarely present with symptoms. When they are present, they are usually associated with local invasion. Often, nonfunctional tumors are diagnosed incidentally during surveillance for other conditions, such as the one established for TSC (25–27). Somatostatin receptor scintigraphy (SRS) and single-photon emission computed tomography (SPECT) are good techniques for initial staging of PNETs with ^68^Ga-labeled somatostatin analogs having the highest sensitivity and specificity for noninsulinoma PNETs and glucagon-like peptide 1 (GLP-1) receptor analogs scintigraphy for patients with insulinomas (24). For well-differentiated grade 1 and 2 that are bigger than 2 cm, growing faster than 0.5 cm in 6–12 month time frame, or Grade 3 is an indication for surgery. mTOR inhibitors seem promising in the medical management of Grade 3 tumors. Surgical resection is indicated in cases of large tumors, rapidly growing or poorly differentiated Grade 3 PNEC (14). For well-differentiated, Grade 1 and 2 nonfunctional PNETs <2 cm and growing <0.5 cm during 6–12 month time frame conservative management is recommended with follow up imaging (CT or MRI); however, prospective studies are needed to further define surgical intervention, as early removal of this lesions may be associated with better long term outcomes (24, 28). The 5-year progression free survival for incidentally diagnosed nonfunctional PNETs is 86%, compared to 59% in symptomatic functional tumors (26).

More than 40 cases of PNETs have been reported in association with TSC. Of those, 21 are functional and 19 are nonfunctional with more than 15 nonfunctional PNET cases being reported after 2012 (7–10, 29–37). Previous case series reported on the presence of both functional and nonfunctional PNETs in association with TSC (9, 34). Extrapolating data from these case series, the estimated prevalence of both functional and nonfunctional PNETs in patients with TSC is 4–9% (9, 34). Previously, a prevalence of 1% of PNETs in association with TSC (38). Since the publication of the 2012 surveillance recommendations, however, there has been a rise in the number of case reports of nonfunctional PNETs in individuals with TSC. As there is a lack of surveillance guidelines for management of nonfunctional PNETs specifically in relation to TSC, individuals may be receiving surgical intervention unnecessarily or earlier than needed based on size and growth rate. Unlike the previously mentioned genetic conditions, the use of mammalian target of rapamycin (mTOR) is indicated for TSC to reduce the size of TSC-related tumors. The use of mTOR inhibitors may be impact the growth or size of nonfunctional PNET growth. Unfortunately, there is limited data available about impact of mTOR inhibitors on TSC- associated PNETs.

This report focuses on nonfunctional PNETs in association with TSC, primarily diagnosed as incidental findings on routine surveillance for renal angiomyolipomas after the establishment of the surveillance guidelines in 2012. As functional PNETs are often diagnosed before being apparent on abdominal MRI and management depends upon presence of symptoms, we did not focus our analysis on these tumors. Our overall objective is to raise awareness and educate clinicians on the emerging pancreatic phenotype observed in the TSC population in the hopes of improved clinical decision making regarding nonfunctional PNETs in TSC, thereby optimizing clinical outcomes that can lead to an improved quality of life. The purposes of our study were to: (1) Clinically characterize nonfunctional PNETs in a large population of patients with TSC, (2) Evaluate the impact of mTOR inhibitors on tumor growth, and (3) Review medical management reported in our case series, in conjunction with the reports in the medical literature, to summarize the current management and treatment regimens for nonfunctional PNETs.

## Materials and Methods

In this case series, we extracted individuals with a diagnosis of TSC and nonfunctional PNET from two separate databases. The study design and data gathering process were developed by the authors and approved by the Institutional Review Board of University of Texas Health Science Center at Houston (HSC-MS-19-0273) and Cincinnati Children's Hospital Medical Center (IRB #2012-2317). Data collection was performed from June 2019–August 2019. For individuals in the case series, each respective clinic site completed our author-designed questionnaire. All data was de-identified prior to being exported to the authors.

### Study Sample

**Figure 1:**
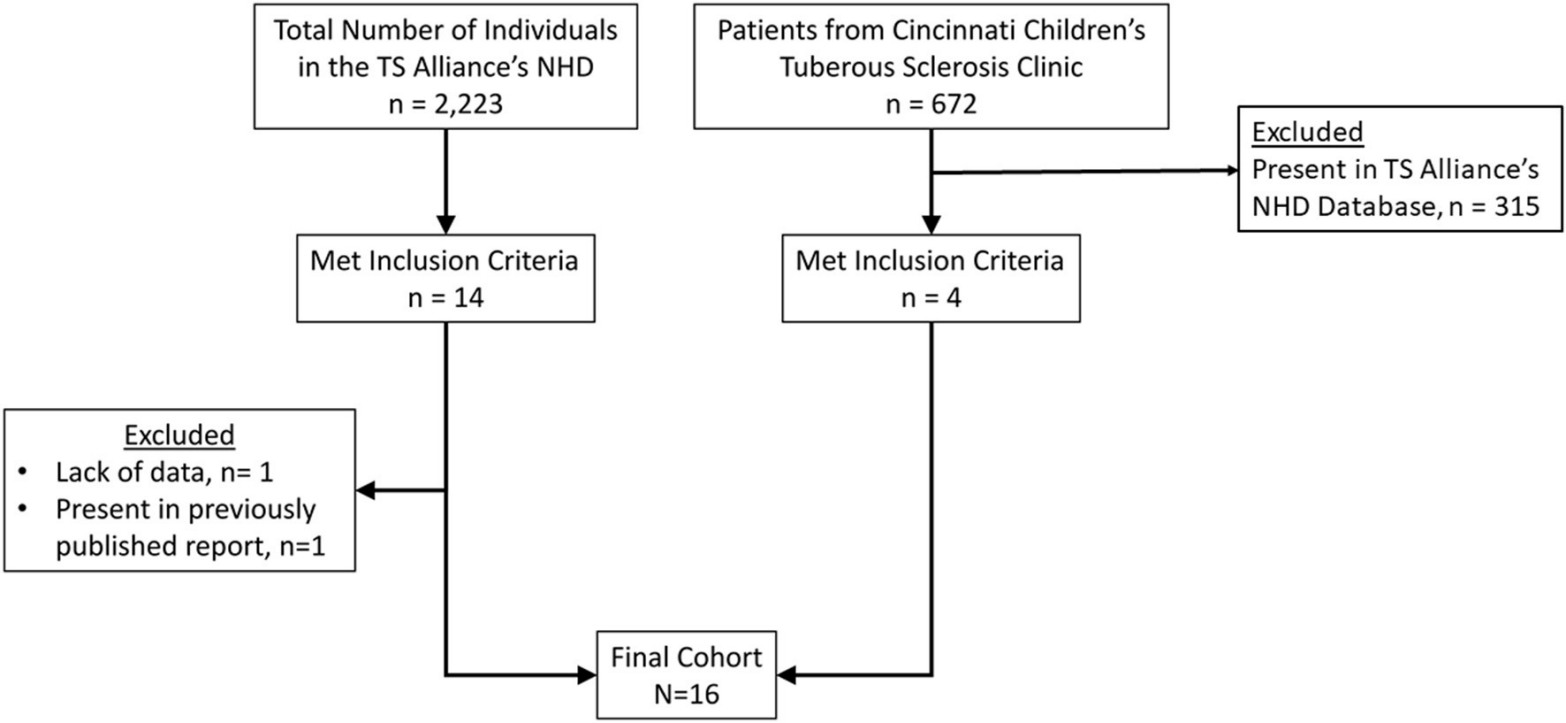
Study flow chart and patient selection process.

Based on the prevalence of TSC (1:11.000) and the anticipated low frequency of PNETs in TSC subjects, participants were obtained through two databases: TS Alliance's Natural History Database and the Cincinnati Children's Hospital TSC Database (Figure 1). Specifically, the TS Alliance's Natural History Database is comprised of individuals with TSC among 18 U.S-based clinical sites. For each database, a single person extracted individuals that met our inclusion criteria. Our inclusion criteria include a clinical and/or molecular diagnosis of TSC as well as the presence of a nonfunctional PNET. Individuals were excluded from our study if they did not have a clinical or molecular diagnosis of TSC, functional PNET, or had a secondary diagnosis of a condition that is associated with an increased risk of PNETs, such as NF-1, VHL, or MEN-1. Once identified as eligible for inclusion, we cross-compared multiple, individual-specific data points between the two clinical databases as well as the medical literature to ensure each subject in the new cohort was unique.

### Data Collection

The questionnaire completed by each respective clinic site included questions regarding supplemental demographics, PNET characteristics, serial imaging of nonfunctional PNETs, as well as previous or current use of a systemic mTOR inhibitor. Specifically, demographic data included current age, sex, age of TSC diagnosis, and clinical vs. molecular diagnosis. PNET data included age of diagnosis, imaging modality that lead to the PNET diagnosis, location of PNET, functionality, number of tumors, and reported clinical vs. surgical management. For available individuals, serial images were obtained for the nonfunctional PNET. All available imaging reports were reviewed to extract the date of evaluation, modality (CT vs. MRI vs. ultrasound) and the diameter of the tumor(s) in mm. Lastly, we obtained the start and stop date for any individual with a history or current use of a systemic mTOR inhibitor.

### Statistical Analysis

Descriptive statistics were performed with categorical data described as frequencies (and percentages) and continuous data described as medians (interquartile ranges (IQR) and ranges). Pearson correlation coefficients was calculated to assess the linear correlation between the nonfunctional PNET size at time of diagnosis of the PNET and patient age. The data from patients that had nonfunctional PNET size information from two or more scans was set up as panel data and analyzed as a longitudinal dataset. Generalized linear mixed models were utilized to identify temporal changes in the size of the PNET (dependent variable) while adjusting for age of the patient, pancreatic location of the PNET and the use of mTOR inhibitors at the time of the scan. Estimates temporal trends were described for the variables along with 95% confidence intervals. Statistical significance was assumed at a Type I error rate of 5%. All analyses were performed in Stata (v.14, College Station, Texas).

## Results

Eighteen TSC patients with nonfunctional PNET were identified in the TS Alliance's Natural History Database (*n* = 14) and the Cincinnati Children's Hospital TSC Database (*n* = 4). We excluded one report from the TS Alliance's Natural History Database (*n* = 13) from the final analysis due to lack of information, but this report was included in the frequency calculation. Furthermore, a duplicated report was identified between the TS Alliance's Natural History Database and the Cincinnati Children's Hospital TSC Database. The duplicated report was removed from the TS Alliance's Natural History Database (*n* = 12) and from the frequency calculation. In our series, 0.65% (17/2,580) of patients have a nonfunctional PNET (Table 1). Table 2 summarizes the 13 publications that describe 19 patients with TSC and nonfunctional PNET.

**Table 1:** Clinical information of subjects with TSC diagnosed with nonfunctional PNET in our case series.

**Patient #**	**TSC1/TSC2**	**Variant**	**Variant classification**	**Age of at PNET Dx (years)**	**Sex**	**Location**	**Diameter at Dx**	**Hx of mTOR**	**Surgery**	**Serial MRI**	**Serial CT**	**Ultrasound**
1	TSC1	9>0insT12956	Pathogenic	8	M	Head	1.0 cm	Yes	No	✓	X	X
2	–	–	–	13	M	Tail	1.4 cm	Yes	No	✓	X	X
3	TSC2	3 bp deletion of AAG	VUS	16	F	Body	0.7 cm	Yes	No	✓	X	X
4	TSC1	c.228 C>T	Pathogenic	3	F	Body	0.7 cm	No	No	✓	X	X
5	TSC2	c.3281C>A	Pathogenic	15	F	Body	1.9 cm	No	No	✓	X	X
6	–	–	–	46	M	Body	Unknown	No	Yes	X	✓	X
7	TSC2	c.4279delA	Pathogenic	12	M	Tail	1.7 cm	No	Yes	✓	X	X
8	TSC2	3 bp deletion of CAT; c.1108C>T	Pathogenic; Benign	21	F	Body	4.1 cm	Yes	Yes	–	–	–
9	TSC1	c.330insT	Pathogenic	7	M	Tail	1.2 cm	No	Yes	✓	✓	X
10	–	–	–	9	F	Tail	1.5 cm	No	Yes	✓	X	✓
11	–	–	–	6	M	Tail	2.0 cm	Yes	Yes	✓	X	X
12	–	–	–	10	F	Body	1.0 cm	Yes	Yes	–	–	–
13	TSC2	c.4646 A>G	Pathogenic	18	M	Body	1.1 cm	No	Yes	✓	X	X
14	–	–	–	15	M	Tail	2.3 cm	No	Yes	–	–	–
15	TSC2	c.5238_5255del18	Pathogenic	9	F	Head	1.6 cm	Yes	No	✓	X	X
16	–	–	–	32	M	Head	3.8 cm	Yes	Unknown	–	–	–

*✓, receiving imaging follow up; X, not receiving imaging follow up; –, no information provided*.

**Table 2:** Clinical information from individuals with TSC diagnosed with nonfunctional PNETs reported in the medical literature.

**Publication**	**TSC1/TSC2**	**Exon/c**.	**Age at PNET Dx**	**Sex**	**Location**	**Single/Multiple**	**Diameter**	**Management**
Ilgren et al. (35)	Unknown	–	23 years	F	–	Single	–	Discovered during autopsy
Verhoef et al. (36)	TSC2	Exon 12	12 years	M	Tail	Single	9.5 cm	Malignant, surgical removal
Francalanci et al. (30)	TSC2	Exon 33	6 years	M	Body/Tail	Single	–	Malignant, surgical removal
Merritt et al. (29)	TSC2	1 bp insertion at position 45-46	39 years	M	Body/Tail	Multiple	–	Surgical removal
Larson et al. (34)	Unknown	–	39 years	M	Tail	Single	4.8 cm	Cystic
	Unknown	–	48 years	M	Tail	Single	3.7 cm	Cystic and enlarged by 25% in 5 years
	Unknown	–	51 years	F	–	–	–	Discovered during autopsy
van den Akker et al. (33)	Unknown	–	–	M	Unknown	Single	–	Surgical removal
Diaz et al. (32)	Unknown	–	31 years	M	Tail	Single	2.3 cm	Surgical removal
Arva et al. (31)	TSC2	Transition A>G in IVS17-2	15 years	M	Body, Tail	Multiple	8.2 cm 1.2 cm	Malignant, surgical removal
Bombardieri et al. (7)	TSC2	c.5160+2_5160+3insT	10 years	M	Head	Single	3.3 cm	Surgical removal
Mortaji et al. (8)	TSC1	Exon 15 c.1530_1531delCA	35 years	F	Tail	Single	1.1 cm	Surgical removal
Koc et al. (9)	Unknown	–	12 years	M	Tail	Single	1 cm	Surgical removal
	Unknown	–	5 years	M	Tail	Single	2.6 cm	Reduced in size on everolimus and then surgical removal
	Unknown	–	19 years	F	Body	Single	2.7 cm	Clinical observation; stable size on everolimus
	Unknown	–	13 years	M	Tail	Single	4.0 cm	Clinical observation; reduced in size on everolimus
	Unknown	–	14 years	M	Tail	Single	0.2 cm	Clinical observation
Mehta et al. (10)	TSC1	Exon 10 c.989dupT	3 years	M	Body	Single	0.4 cm	Surgical removal at 1 cm
Amarjothi et al. (11)	Unknown	–	17 years	F	Head	Single	2.5 cm	Surgical removal

### Demographics

At the time of data extraction, the median age was 18.0 years (range 3–55 years, IQR: 15.5–25). Table 3 shows the demographics, age of PNET diagnosis, and method of diagnosis in the case series and the individuals reported in the medical literature with a nonfunctional PNET and TSC. In the case series, sex was relatively equal with 56.3% (*n* = 9) male and 43.7% (*n* = 7) female. The age of TSC diagnosis was available for 14 of the patients. Most (*n* = 9, 64%) were diagnosed with TSC at <1 year of age with a median age of diagnosis of 8.5 months (range: 1 month to 7 years; IQR 3.5 months to 1 year). Results from genetic testing were available for just over half (56.3%, *n* = 9) of the patients. Information on *TSC1* and *TSC2* variants is in Table 1.

**Table 3:** Demographic information of patients in this case series and published case reports.

	**Frequency, *n* (%)^†^**	
	**Case series**	**Reported cases**
	**(*n* = 16)**	**(*n* = 19)**
**Age at data collection**		
≤19 years	10 (62.5)	62.5 (11)
20–39 years	4 (25)	25 (5)
40–59 years	2 (12.5)	12.5 (2)
**Sex**
Male	9 (56.3)	56.3 (14)
Female	7 (43.7)	43.7 (5)
**Age at TSC diagnosis**		
0–11 months	8 (57.1)	57.1 (2)
1–3 years	5 (31.3)	31.3 (2)
≥4 years	1 (6.2 )	6.2 (4)
Unknown	2 (12.5)	12.5 (11)
**Molecular diagnosis of TSC**		
Yes	8 (50.0)	50.0 (7)
No or unknown	8 (50.0)	50.0 (12)
Age at PNET diagnosis, years, median (IQR)^*^	12.5 (8.5 - 17)	16 (12 - 34)
**Age of PNET diagnosis**		
≤10 years	7 (43.8)	43.8 (4)
11–20 years	6 (37.5)	37.5 (7)
21–30 years	1 (6.2)	6.2 (1)
31–40 years	1 (6.2 )	6.2 (4)
≥41 years	1 (6.2 )	6.2 (2)
**Initial diagnosis method**		
MRI	9 (56.3)	56.3 (8)
CT	2 (12.5)	12.5 (5)
Ultrasound	4 (25.0)	25.0 (3)
Other	1 (6.2 )	6.2 (2)

† Unless otherwise stated;

* *IQR, interquartile range*.

**Figure 2:**
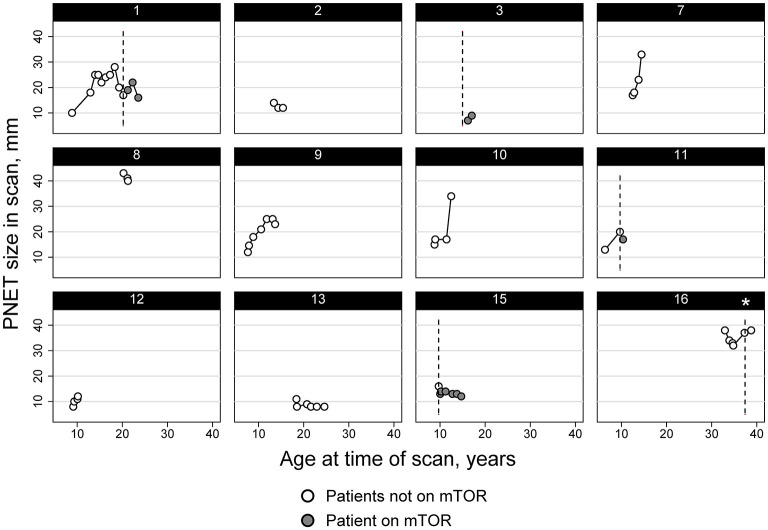
Diameter trend of PNET in subjects from the TS Alliance's Natural History Database and the Cincinnati Children's Hospital TSC Database. Asterisk indicates that Subject 16 started and stopped an mTOR inhibitor therapy between imaging and never had any imaging performed while actively taking an mTOR inhibitor.

In the case series, half of the individuals (*n* = 8) had a history of treatment with a systemic mTOR inhibitor (oral everolimus or sirolimus). Of those, 63% (*n* = 5) used a systemic mTOR inhibitor during the time of image acquisition in which the nonfunctional PNET was identified and/or followed serially (Figure 2).

### Genetic Testing Results

Just over half (56.3%, *n* = 9) of the patients provided results from genetic testing and 43.7% (*n* = 7) either did not undergo genetic testing and/or did not provide results from genetic testing (Table 1). Of the nine individuals who provided results from genetic testing, four variants were found in *TSC1*, and six variants were identified in *TSC2*. There was one individual who was found to have a one pathogenic variant and one benign variant in *TSC1*. Of the 4 variants identified in *TSC1*, three were classified as pathogenic and one was classified as benign. Of the six variants identified in *TSC2*, five were classified as pathogenic and one was classified as a variant of uncertain significance (VUS).

### Imaging Modality of PNET Identification

**Figure 3:**
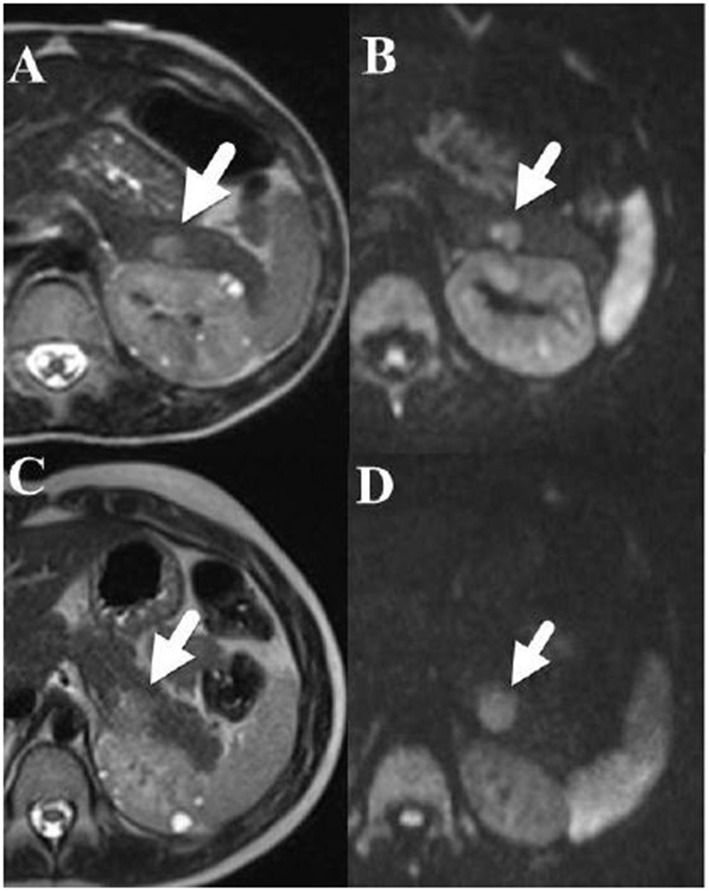
**(A)** [Upper left] Axial T2-weighted fast spin echo and **(B)** [Upper right] diffusion weighted MRI images performed in a 6-year-old boy show a 1.2 × 0.8 cm hyperintense mass (arrow) in the tail of the pancreas that restricts diffusion. Note that small cysts are also present within the left kidney. **(C)** [Lower left] an Axial T2-weighted fast spin echo and **(D)** [Lower right] diffusion weighted MRI images performed 3 years later show that the pancreatic tail mass (arrow) has grown slowly and now measures 1.4 × 1.5 cm.

In the case series, the median age of nonfunctional PNET diagnosis was 12.5 years with a range from 3 to 46 years of age (IQR: 8.5–17) (Table 3). Most individuals (93.8%, *n* = 15) had their tumors incidentally identified on routine imaging. MRI was the modality on which PNET was more commonly identified (*n* = 9, 56.3%), followed by ultrasound (*n* = 4, 25%) and CT (*n* = 2, 12.5%). One individual's tumor was diagnosed during a distal pancreatectomy and splenectomy. The location of the PNET was 43.8% (*n* = 7) in the body of the pancreas, 37.5% (*n* = 6) in the tail of the pancreas, and 18.7% (*n* = 3) in the head of the pancreas. Of note, the diagnosis of the nonfunctional PNET was delayed in 3 patients. Retrospective review of prior imaging revealed the presence of the nonfunctional PNET on a previous imaging series. In these 3 cases, the sizes of the missed PNETs were 8 mm on MRI (diagnosed a year later at 11 mm on MRI), 13 mm on MRI (diagnosed 3 years later at 15 mm on MRI) and 43 mm on MRI (diagnosed a year later at 41 mm on CT). Figures 3A–D represents the initial and subsequent abdominal MRIs of a nonfunctional PNET that was not diagnosed on the original imaging.

### Follow-Up Management and Tumor Growth

Over half of the individuals (*n* = 9) underwent surgical intervention for their nonfunctional PNET. Surgical status was unknown for one patient. Due to the retrospective nature of this study, the clinical indication for the surgeries was not reported to the authors. Clinical imaging follow-up was available for 12 patients. Of these patients, six individuals did not undergo surgical intervention and only had serial imaging follow-up (Table 1). MRI was used for follow-up imaging in all but one patient. CT was solely utilized in one subject and ultrasound was utilized for follow-up for only one patient but was used in combination with MRI.

**Figure 4:**
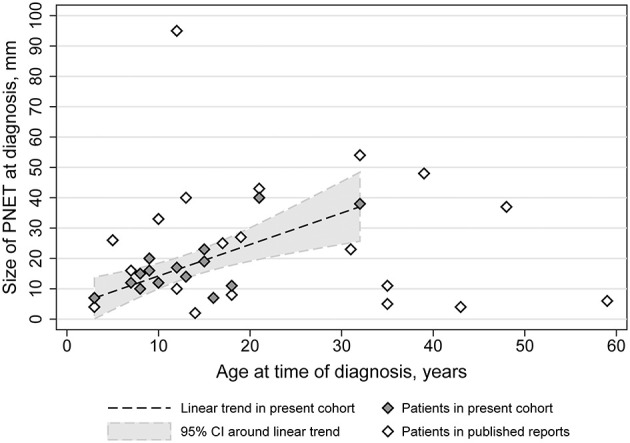
PNET diameter as a function of age in TSC subjects of the TS Alliance's Natural History Database, the Cincinnati Children's Hospital TSC Database, and previously published cases.

In the case series, data on the size of the nonfunctional PNET at the time of diagnosis was available for 15 patients. The median size of the PNET at that time was 15 mm (interquartile range: 11–19 mm; range: 7–40 mm). There was a strong positive correlation (ρ = 0.74, 95% confidence interval = 0.37–0.91; *p* = 0.002) between the age at diagnosis of the PNET and its size in our cohort (Figure 4). A linear mixed model of our cohort data suggested that for every year increase in the age of diagnosis, the PNET size at the time of diagnosis was increased by 1.04 mm (95% confidence interval = 0.47–1.60 mm).

Twelve of the sixteen total patients had two or more imaging studies performed where the PNET could be visualized and measured longitudinally. The average rate of change of the PNET was an increase of 2.0 mm/year but varied considerably from person to person and even within a person over time (Figure 2). Overall, the median rate of change per patient was 1.02 mm/year (IQR: 0.0–5.02) and ranged from a decrease in size of 5.7 mm/year to an increase of 13 mm/year, with standard deviations for these individual patient size changes ranging from 1.4 to 11.0 mm/year. Panel data analysis using mixed models that adjusted for age of the patient and use of mTOR inhibitors, identified an independent effect of time on the size of the PNET, with an average increase of 0.95 mm per year (95% CI: 0.54–1.36 mm). When adjusted for the age of the patient and location of the PNET, the mixed models also demonstrated an association between mTOR use and the size of the PNET with patients on mTOR inhibitors having PNETs that were smaller compared to the size measured in patients not taking mTOR inhibitors (difference of 5.5 mm, 95% CI: 2.1–9.0 mm) (Figure 2).

We had information on 12 subjects regarding tumor growth over time. Of those, five were on systemic mTOR therapy. Three demonstrated a decrease in the tumor size (subjects 1, 11, and 15), one was stable (subject 16), and one demonstrated an increase in tumor size (subject 3). Of the 7 without any mTOR treatment, 4 showed a spontaneous decrease in tumor size (2, 8, 9, and 13) at some points in time but growth at other time points. Tumor growth rate was slightly less in individuals taking an mTOR inhibitor (−0.8 mm/yr, IQR: −2.3 to 2.2) than those without (1.6 mm/yr; IQR: −0.99 to 5.01) but the difference was not statistically significant (*p* > 0.05).

## Discussion

This case series reports 16 new cases of nonfunctional PNETs in association with TSC. New cases were identified from the TS Alliance's Natural History Database that follows 2,223 TSC subjects across the United States and from Cincinnati Children's Hospital TSC Database that follows an additional 357 subjects. We documented rate of growth, age of diagnosis of nonfunctional PNETs, location, management and growth rate in the presence and absence of mTOR inhibitors. Our study adds to the growing literature of reported nonfunctional PNETs in hopes to encourage the creation of consensus guidelines and utilization of mTOR inhibitor therapy for this rare clinical feature of TSC.

### Frequency

The number of reported functional and nonfunctional PNETs associated with TSC has increased over the last decade, likely coinciding with 2012 consensus recommendations for abdominal MR imaging every 1–3 years to detect and monitor renal angiomyolipoma (2). Indeed, nearly all our cases were diagnosed as unexpected findings during recommended surveillance. The estimated frequency of nonfunctional PNET in our case series is 0.65%. The reported prevalence in the general population of 0.003% (15, 16, 39). Whereas, the prevalence reported for PNET in association with other genetic syndromes such as MEN-1, VHL, and NF-1 is 80%, 9–17%, and <10%, respectively (18–22, 40).

### Delayed Diagnosis

Prior single center TSC studies have estimated PNET prevalence of nonfunctional and functional PNETs to be between 4 and 9% (9, 34). The TS Alliance's Natural History Database and the Cincinnati Children's Hospital TSC Database utilized in our study provided a starting cohort much larger in size and representative of patients with TSC across many different TSC centers in the United States, but did have the limitation that identification of PNET cases in our study depended on numerous outside radiologists' recognition and reporting of PNET in final imaging reports rather than study-specific review of the individual's imaging. As a result, our study identified that 3/16 (19%) tumors in our cohort were retrospectively found to be present on prior imaging but were not identified or reported initially. The tumor diameter differences between measurements was larger than MRI spatial resolution. Therefore, the frequency in our study likely underestimates true frequency of nonfunctional PNET in TSC. Under-recognition of PNETs is important to acknowledge, as pancreatic findings are not commonly expected in TSC, and the primary purpose for abdominal MRI is for diagnosis and surveillance of renal findings rather than pancreatic. Under these circumstances, radiologists may miss small pancreatic tumors or misdiagnose them as being pancreatic angiomyolipomas, which have been reported in association with TSC (41). This information particularly highlights the importance of paying special attention to the pancreas on surveillance abdominal MRIs of individuals with TSC and compare to previous images.

### Age of Nonfunctional PNET Diagnosis

Younger individuals with TSC included in our cohort were more likely to have abdominal MRIs in childhood given their current age in relation with the establishment of the surveillance guidelines in 2012. Given this, it is possible that the older individuals with TSC could have received their diagnosis of a nonfunctional PNET earlier through what is now standardized surveillance recommendations. Even when considering this limitation, PNETs in the general population occur with peak prevalence between 70–80 years of age and are highly associated with malignancy. Surprisingly, the average age of PNET in TSC appears to be young (42, 43). The median age in our cohort was 18 years at diagnosis, with 88% under the age of 40 years and no one diagnosed older than 60 years. Prior published cases demonstrate similar age prevalence (Table 3). Also, it is notable that none of the previously mentioned genetic syndromes have surveillance protocols with abdominal imaging for individuals younger than 16 years of age; therefore, their PNET prevalence may be under-estimated due to lack of surveillance guidelines for younger subjects in comparison to individuals with TSC. In comparable studies of nonfunctional PNETs associated with MEN-1 diagnosed by endoscopic ultrasonography, the mean age of diagnosis is 30 years (range: 13–65 years) and reports that 18% of MEN-1 males had a diagnosis by 20 years of age (42, 43).

### Genotype-Phenotype Correlations

In this study, it was difficult to arrive at conclusions on genotype-phenotype correlations in relation to nonfunctional PNETs due to the limited size of this case series. Genotype information was available in nine subjects. Six had *TSC2* pathogenic variants and 3 had *TSC1* pathogenic variants (Table 1). In previous publications, molecular diagnosis was available in 7 out of the 19 cases (Table 2). Five cases were reported having pathogenic variants in *TSC2* and two cases had *TSC1* pathogenic variants. Based on the data available from previous publications and our case series, the proportion of nonfunctional PNET cases due to *TSC2* compared to *TSC1* reflects the mutation spectrum described in TSC rather than suggests an increased occurrence of nonfunctional PNETs with *TSC2* pathogenic variants. Furthermore, there was not any correlation between location or type of pathogenic variants with the likelihood of development or clinical characteristics of nonfunctional PNETs in our series or previously reported cases.

### Management of Nonfunctional PNETs

It is difficult to ascertain if the development of TSC-associated nonfunctional PNETs is an age-related phenomenon that eventually will resolve as many individuals in our cohort had their nonfunctional PNET surgically removed. The current surgical recommendations for sporadic PNETs are to only remove tumors over 20 mm in size or that are doubling faster (24, 44). In our cohort of patients, we documented reduction of the size of the tumors in several individuals over time, but there are no reports of fully self-resolved pancreatic TSC-associated nonfunctional PNETs after introduction of surveillance protocols. This unresolved issue becomes particularly relevant for TSC, a condition for which resolution of neonatal cardiac rhabdomyosarcomas is routinely observed and lack of malignant transformation for most TSC-related tumors (30, 36). In our cohort, there were no instances of malignant nonfunctional PNETs, but they have been three separate case reports by Francalanci et al. (30), Arva et al. (31), and Verhoef et al. (36) that have documented this occurrence. All three cases were males from 6 to 15 years old with germline *TSC2* pathogenic variants. Two of these cases documented loss of heterozygosity (LOH) in tumor tissue (30, 36). Based on the rare occurrence of PNET and the predominance of *TSC2* cases, it is difficult to conclude on specific associations between *TSC2* and male-sex as risk factors for malignant PNETs. Like sporadic PNETs, the recognition of malignant PNETs in association with TSC supports the recommendations of the PNET guidelines for long-term clinical follow up and intend surgical intervention for tumors larger than 2.5 cm in diameter or rapidly growing tumors (24, 44).

Interestingly, we had one documented recurrence of nonfunctional PNET that was confirmed by the clinic site to the authors. This instance occurred 4 years after resection of the initial tumor. To the best of our knowledge, there are no other reported cases of recurrent nonfunctional PNETs in association with TSC. This highlights the importance of continuing the surveillance for these tumors despite initial resection.

There are no reports in the medical literature of functional transformation of nonfunctional PNETs in association with TSC. Additionally, we have not seen this phenomenon documented in the TS Alliance's Natural History Database or Cincinnati Children's Hospital TSC Database. However, there are six reports of transformation of nonfunctional PNETs into functional in cases of sporadic PNETs (18, 45–48). Nahmias et al. (47) described on three adults, two with nonfunctional PNETs and one with a gastrinoma. All three individuals progressed to insulinomas. Two of these cases, the individual with the gastrinoma and one with the nonfunctional PNET, demonstrated tumor reduction with the use of everolimus for a short period of time, but eventually required surgical removal. Sayki Arslan et al. (48) reported on a PNET measuring 3.1 × 2.7 cm that transformed into a malignant insulinoma in a 62-year-old male. His diagnosis of nonfunctional PNET occurred 3 years before the transformation into an insulinoma. Functional transformation remains a possibility for PNET in association with a TSC diagnosis. Interestingly, these publications provide additional data that argues in favor for the use of mTOR inhibitor treatment for PNETs associated with TSC, in particular for functional PNETs.

### Tumor Growth and mTOR Inhibitor Therapy

We had information on 12 subjects regarding tumor growth over time. Of those, five were on systemic mTOR therapy during varying portions of the study period. It is well-known that mTOR inhibitors have showed reduction in TSC-related tumors across many organ systems. The tumor growth rate was slightly less in individuals taking an mTOR inhibitor than those without. Although, our case-series was not powered for this comparison and the difference was not statistically significant, the trend observed was consistent with the reported effect of mTOR inhibitors on PNET growth in TSC and is the basis for the recommendation of its use on advanced PNETs (49). To better understand the natural history of these tumors and their response to mTOR treatment, prospective studies are needed in TSC with standardized imaging paradigms with special attention to potential confounding factors regarding tumor growth.

### Study Limitations

This descriptive report is a case series, and as such wasn't powered for comparative analyses, especially for comparisons to other studies. The few comparisons we did perform within our cohort (e.g., tumor growth rate in patients on mTOR inhibitors and those without) were exploratory in nature, and are presented as such. Although the analyses, including the adjusted mixed regression models, are appropriate means of more thoroughly describing our cohort, it should be noted that due to the exploratory nature we have not performed any other statistical corrections for multiple testing. Additionally, our study design does not allow for full recognition of the presence of age-related penetrance of PNETs in TSC and the natural history of these tumors. Additionally, the lack of uniformity across radiologists reading the images and modalities used could introduce measurement error regarding the nonfunctional PNETs size. Lastly, the source of our study sample was composed of individuals followed at specialized centers across multiple clinical sites in the United States, who voluntarily chose to participate in large natural history databases. This might result in a selection bias due to exclusion of individuals with milder disease.

### Conclusions

Based on our data, TSC-associated nonfunctional PNETs are slow growing, and the majority appear to be benign or nonmalignant in nature. This study provides preliminary evidence supporting the use of mTOR inhibitor therapy in conjunction with serial imaging as medical management for nonfunctional PNETs as an alternative option to invasive surgical removal. This can easily be integrated into the TSC surveillance recommendations for abdominal MRIs every 1–3 years for monitoring of angiomyolipomas and renal cysts (2). Furthermore, patients with TSC often have other indications for the use of mTOR inhibitors, such as large angiomyolipomas, SEGAs, lymphangioleiomyomatosis, or refractory epilepsy. As described here, mTOR inhibitors may slow the rate of growth of nonfunctional PNETs, but it remains unanswered if the use of mTOR inhibitors should be the initial method of medical management for nonfunctional PNETs or an alternative to surgical removal. Other unresolved issues include the possibility of self-resolution or associated risk factors for these tumors including genotype-phenotype associations, age-related penetrance, and rate of malignancy. Only then will solid evidence-based surveillance and treatment recommendations be possible for nonfunctional PNET occurring in the setting of TSC.

## Data Availability Statement

The raw data supporting the conclusions of this article will be made available by the authors, without undue reservation.

## Ethics Statement

The studies involving human participants were reviewed and approved by Institutional Review Board of University of Texas Health Science Center at Houston and Cincinnati Children's Hospital Medical Center. Written informed consent from the participants' legal guardian/next of kin was not required to participate in this study in accordance with the national legislation and the institutional requirements.

## Author Contributions

KM and DR-B: conceptualization and study design, manuscript writing, data collection, statistical analysis, and manuscript editing. HN: conceptualization and study design, data review, and manuscript editing. PR: manuscript writing, data collection, statistical analysis, and manuscript editing. SH: statistical analysis and manuscript editing. DK, JC, ATT, and AJT: data collection, data review for Cincinnati subjects, and manuscript editing. DE-F: data collection for Cincinnati subjects and critical revision of the manuscript. DF: data collection and data review for Cincinnati subjects. All authors contributed to the article and approved the submitted version.

## Conflict of Interest

The authors declare that the research was conducted in the absence of any commercial or financial relationships that could be construed as a potential conflict of interest.

## References

[1] Ebrahimi-Fakhari D Mann LL Poryo M Graf N von Kries R Heinrich B. Incidence of tuberous sclerosis and age at first diagnosis: new data and emerging trends from a national, prospective surveillance study. Orphanet J Rare Dis. (2018) 13:1–8. 10.1186/s13023-018-0870-y 30016967 PMC6050673

[2] Krueger DA Northrup H. Tuberous sclerosis complex surveillance and management: recommendations of the 2012 International Tuberous Sclerosis Complex Consensus Conference. Pediatr Neurol. (2013) 49:255–65. 10.1016/j.pediatrneurol.2013.08.002 24053983 PMC4058297

[3] Sancak O Nellist M Goedbloed M Elfferich P Wouters C Maat-Kievit A. Mutational analysis of the TSC1 and TSC2 genes in a diagnostic setting: genotype-phenotype correlations and comparison of diagnostic DNA techniques in Tuberous Sclerosis Complex. Eur J Hum Genet. (2005) 13:731–41. 10.1038/sj.ejhg.5201402 15798777

[4] The European Chromosome 16 Tuberous Sclerosis Consortium. Identification and characterization of the tuberous sclerosis gene on chromosome 16. Cell. (1993) 75:1305–15. 10.1016/0092-8674(93)90618-Z 8269512

[5] van Slegtenhorst M de Hoogt R Hermans C Nellist M Janssen B Verhoef S. Identification of the tuberous sclerosis gene TSC1 on chromosome 9q34. Science. (1997) 277:805–8. 10.1126/science.277.5327.805 9242607

[6] Au KS Williams AT Roach ES Batchelor L Sparagana SP Delgado MR. Genotype/phenotype correlation in 325 individuals referred for a diagnosis of tuberous sclerosis complex in the United States. Genet Med. (2007) 9:88–100. 10.1097/GIM.0b013e31803068c7 17304050

[7] Bombardieri R Moavero R Roberto D Cerminara C Curatolo P. Pancreatic neuroendocrine tumor in a child with a tuberous sclerosis complex 2 (TSC2) mutation. Endocr Pract. (2013) 19:e124–8. 10.4158/EP13010.CR 23757617

[8] Mortaji P Morris KT Samedi V Eberhardt S Ryan S. Pancreatic neuroendocrine tumor in a patient with a TSC1 variant: case report and review of the literature. Familial Cancer. (2018) 17:275–80. 10.1007/s10689-017-0029-3 28887784

[9] Koc G Sugimoto S Kuperman R Kammen BF Karakas SP. Pancreatic tumors in children and young adults with tuberous sclerosis complex. Pediatr Radiol. (2017) 47:39–45. 10.1007/s00247-016-3701-0 27639993

[10] Mehta S Rusyn L Ginsburg H Hajdu C Kohn B. Pancreatic neuroendocrine tumor in a young child with tuberous sclerosis complex 1. J Endocr Soc. (2019) 3:1201–6. 10.1210/js.2019-00051 31187078 PMC6546344

[11] Amarjothi JMV Jesudason J Ramasamy V Babu OLN. Interesting pancreatic tumour in the background of tuberous sclerosis. BMJ Case Rep. (2019) 12:e227292. 10.1136/bcr-2018-227292 31383671 PMC6685411

[12] Halfdanarson TR Rabe KG Rubin J Petersen GM. Pancreatic neuroendocrine tumors (PNETs): incidence, prognosis and recent trend toward improved survival. Ann Oncol. (2008) 19:1727–33. 10.1093/annonc/mdn351 18515795 PMC2735065

[13] Nagtegaal ID Odze RD Klimstra D Paradis V Rugge M Schirmacher P. The 2019 WHO classification of tumours of the digestive system. Histopathology. (2020) 76:182–8. 10.1111/his.13975 31433515 PMC7003895

[14] Khanna L Prasad SR Sunnapwar A Kondapaneni S Dasyam A Tammisetti VS. Pancreatic neuroendocrine neo-plasms: 2020 update on pathologic and imaging findings and classification. Radiographics. (2020) 40:1240–62. 10.1148/rg.2020200025 32795239

[15] Lawrence B Gustafsson BI Chan A Svejda B Kidd M Modlin IM. The epidemiology of gastroenteropancreatic neuroendocrine tumors. Endocrinol Metab Clin North Am. (2011) 40:1–18. 10.1016/j.ecl.2010.12.005 21349409

[16] Yao JC Hassan M Phan A Dagohoy C Leary C Mares JE. One hundred years after “carcinoid:” epidemiology of and prognostic factors for neuroendocrine tumors in 35,825 cases in the United States. J Clin Oncol. (2008) 26:3063–72. 10.1200/JCO.2007.15.4377 18565894

[17] Metz DC Jensen RT. Gastrointestinal neuroendocrine tumors: pancreatic endocrine tumors. Gastroenterology. (2008) 135:1469–92. 10.1053/j.gastro.2008.05.047 18703061 PMC2612755

[18] Giusti F Marini F Brandi ML. Multiple endocrine neoplasia type 1. In: Adam MP Ardinger HH Pagon RA Wallace SE Bean LJH Stephens K Amemiya A editors. GeneReviews^®^. Seattle, WA: University of Washington, Seattle (2005).

[19] Nishi T Kawabata Y Hari Y Imaoka H Ishikawa N Yano S. A Case of Pancreatic Neuroendocrine Tumor in a Patient with Neurofibromatosis-1. (2012). Available online at: http://www.wjso.com/content/10/1/153 (accessed May 18, 2020). 10.1186/1477-7819-10-153 PMC3545965 22824559

[20] Jensen RT Berna MJ Bingham DB Norton JA. Inherited pancreatic endocrine tumor syndromes: advances in molecular pathogenesis, diagnosis, management, and controversies. Cancer. (2008) 113:1807–43. 10.1002/cncr.23648 18798544 PMC2574000

[21] Alexakis N Connor S Ghaneh P Lombard M Smart HL Evans J. Hereditary pancreatic endocrine tumours. Pancreatology. (2004) 4:417–35. 10.1159/000079616 15249710

[22] van Leeuwaarde RS Pieterman CRC Bleiker EMA Dekkers OM van der Horst-Schrivers AN Hermus AR. High fear of disease occurrence is associated with low quality of life in patients with multiple endocrine neoplasia type 1: results from the Dutch MEN1 Study Group. J Clin Endocrinol Metab. (2018) 103:2354–61. 10.1210/jc.2018-00259 29618015

[23] Young K Iyer R Morganstein D Chau I Cunningham D Starling N. Pancreatic neuroendocrine tumors: a review. Future Oncol. (2015) 11:853–64. 10.2217/fon.14.285 25757686

[24] Falconi M Eriksson B Kaltsas G Bartsch DK Capdevila J Caplin M. E-mail ENETS consensus guidelines ENETS consensus guidelines update for the management of patients with functional pancreatic neuroendocrine tumors and non-functional pancreatic neuroendocrine tumors. Neuroendocrinology. (2016) 103:153–71. 10.1159/000443171 26742109 PMC4849884

[25] Vagefi PA Razo O Deshpande V McGrath DJ Lauwers GY Thayer SP. Evolving patterns in the detection and outcomes of pancreatic neuroendocrine neoplasms: The Massachusetts General Hospital experience from 1977 to 2005. Archiv Surg. (2007) 142:347–53. 10.1001/archsurg.142.4.347 17438169 PMC3979851

[26] Cheema A Weber J Strosberg JR. Incidental detection of pancreatic neuroendocrine tumors: an analysis of incidence and outcomes. Ann Surg Oncol. (2012) 19:2932–6. 10.1245/s10434-012-2285-7 22350605

[27] Kuo JH Lee JA Chabot JA. Nonfunctional pancreatic neuroendocrine tumors. Surg Clin North Am. (2014) 94:689–708. 10.1016/j.suc.2014.02.010 24857584

[28] Partelli S Ramage JK Massironi S Zerbi A Kim HB Niccoli P. Management of asymptomatic sporadic nonfunctioning pancreatic neuroendocrine neoplasms (ASPEN) ≤2 cm: study protocol for a prospective observational study. Front Med. (2020) 7:598438. 10.3389/fmed.2020.598438 33425946 PMC7785972

[29] Merritt JL Davis DME Pittelkow ME Babovic-Vuksanovic D. Extensive acrochordons and pancreatic islet-cell tumors in tuberous sclerosis associated with TSC2 mutations. Am J Med Genet Part A. (2006) 140:1669–72. 10.1002/ajmg.a.31351 16835931

[30] Francalanci P Diomedi-Camassei F Purificato C Santorelli M Giannotti A Dominici C. Malignant pancreatic endocrine tumor in a child with tuberous sclerosis. Am J Surg Pathol. (2003) 27:1386–9. 10.1097/00000478-200310000-00012 14508401

[31] Arva NC Pappas JG Bhatla T Raetz EA Macari M Ginsburg HB. Well-differentiated pancreatic neuroendocrine carcinoma in tuberous sclerosis–case report and review of the literature. Am J Surg Pathol. (2012) 36:149–53. 10.1097/PAS.0b013e31823d0560 22173120

[32] Díaz DD Ibarrola C Sanz RG Hurtado BP Tabares JS Ruizdelgado FC. Neuroendocrine tumor of the pancreas in a patient with tuberous sclerosis: A case report and review of the literature. Int J Surg Pathol. (2012) 20:390–5. 10.1177/1066896911428735 22169969

[33] van den Akker M Angelini P Taylor G Chami R Gerstle JT Gupta A. Malignant pancreatic tumors in children: a single-institution series. J Pediatr Surg. (2012) 47:681–7. 10.1016/j.jpedsurg.2011.11.046 22498381

[34] Larson A Hedgire S Deshpande V Stemmer-Rachamimov A Harisinghani M Ferrone C. Pancreatic neuroendocrine tumors in patients with tuberous sclerosis complex Nothing to declare. Clin Genet. (2012) 82:558–63. 10.1111/j.1399-0004.2011.01805.x 22035404

[35] Ilgren EB Westmorelandt D. Tuberous sclerosis: unusual associations in four cases. J Clin Pathol. (1984) 37:272–8. 10.1136/jcp.37.3.272 6142060 PMC498698

[36] Verhoef S van Diemen-Steenvoorde R Akkersdijk WL Bax NMA Ariyurek Y Hermans CJ. Malignant pancreatic tumour within the spectrum of tuberous sclerosis complex in childhood. Eur J Pediatr. (1999) 158:284–7. 10.1007/s004310051073 10206124

[37] Dworakowska D Grossman AB. Are neuroendocrine tumours a feature of tuberous sclerosis? A systematic review. Endocr Relat Cancer. (2009) 16:45–58. 10.1677/ERC-08-0142 18978035

[38] Eledrisi MS Stuart CA Alshanti M. Insulinoma in a patient with tuberous sclerosis: is there an association? Endocrine Practice. (2002) 8:109–12. 10.4158/ep.8.2.109 11942775

[39] Dasari A Shen C Halperin D Zhao B Zhou S Xu Y. Trends in the incidence, prevalence, and survival outcomes in patients with neuroendocrine tumors in the United States. JAMA Oncol. (2017) 3:1335–42. 10.1001/jamaoncol.2017.0589 28448665 PMC5824320

[40] de Mestier L Gaujoux S Cros J Hentic O Vullierme MP Couvelard A. Long-term prognosis of resected pancreatic neuroendocrine tumors in von Hippel-Lindau disease is favorable and not influenced by small tumors left in place. Ann Surg. (2015) 262:384–8. 10.1097/SLA.0000000000000856 25185468

[41] Sato K Ueda Y Tachibana H Miyazawa K Chikazawa I Kaji S. Malignant epithelioid angiomyolipoma of the kidney in a patient with tuberous sclerosis: an autopsy case report with p53 gene mutation analysis. Pathol Res Pract. (2008) 204:771–7. 10.1016/j.prp.2008.04.008 18547741

[42] Thomas-Marques L Murat A Delemer B Penfornis A Cardot-Bauters C Baudin E. Prospective endoscopic ultrasonographic evaluation of the frequency of nonfunctioning pancreaticoduodenal endocrine tumors in patients with multiple endocrine neoplasia type 1. Am J Gastroenterol. (2006) 101:266–73. 10.1111/j.1572-0241.2006.00367.x 16454829

[43] Triponez F Dosseh D Goudet P Cougard P Bauters C Murat A. Epidemiology data on 108 MEN 1 patients from the GTE with isolated nonfunctioning tumors of the pancreas. Ann Surg. (2006) 243:265–72. 10.1097/01.sla.0000197715.96762.68 16432361 PMC1448903

[44] Kunz PL Reidy-Lagunes D Anthony LB Bertino EM Brendtro K Chan JA. Consensus Guidelines for the Management and Treatment of Neuroendocrine Tumors. (2013). Available online at: www.pancreasjournal.com 10.1097/MPA.0b013e31828e34a4 PMC4304762 23591432

[45] Vashi PG Gupta D Dahlk S. A unique case of a nonfunctional metastatic pancreatic neuroendocrine tumor transforming into an insulin-secreting tumor with an unusual clinical course. Pancreas. (2011) 40:781–4. 10.1097/MPA.0b013e318212c42d 21673539

[46] Koshy AA Gordon IO van Ha TG Kaplan EL Philipson LH. Metastatic insulinoma following resection of nonsecreting pancreatic islet cell tumor. J Investig Med High Impact Case Rep. (2013) 1:232470961247327. 10.1177/2324709612473274 26425568 PMC4528785

[47] Nahmias A Grozinsky-Glasberg S Salmon A Gross DJ. Pancreatic neuroendocrine tumors with transformation to insulinoma: an unusual presentation of a rare disease. Endocrinol Diabetes Metab Case Rep. (2015) 2015:150032. 10.1530/edm-15-0032 26113980 PMC4477235

[48] Sayki Arslan M Ozbek M Karakose M Tutal E Ucan B Yilmazer D. Transformation of nonfunctioning pancreatic tumor into malignant insulinoma after 3 years: an uncommon clinical course of insulinoma. Arch Endocrinol Metab. (2015) 59:270–2. 10.1590/2359-3997000000049 26154097

[49] Lamberti G Brighi N Maggio I Manuzzi L Peterle C Ambrosini V. The role of mTOR in neuroendocrine tumors: future cornerstone of a winning strategy? Int J Mol Sci. (2018) 19:747. 10.3390/ijms19030747 29509701 PMC5877608

